# Transplantation of gingiva-derived mesenchymal stem cells ameliorates collagen-induced arthritis

**DOI:** 10.1186/s13075-016-1160-5

**Published:** 2016-11-11

**Authors:** Yongchun Gu, Songtao Shi

**Affiliations:** 1 Department of Dentistry, First People’s Hospital of Wujiang Dist., Nantong University, Suzhou, China; 2 Center for Craniofacial Molecular Biology, Ostrow School of Dentistry, University of Southern California, 2250 Alcazar Street, CSA 103, Los Angeles, CA USA

**Keywords:** Rheumatoid arthritis, Gingival mesenchymal stem cells, Cell therapy, FasL/Fas pathway

## Abstract

**Background:**

Rheumatoid arthritis (RA) is a chronic, progressive, and inflammatory autoimmune disease which primarily affects the small arthrodial joints. The aim of this study was to test whether transplantation of mesenchymal stem cells derived from gingival tissue (GMSCs) could ameliorate collagen-induced arthritis (CIA), and to explore the role of the FasL/Fas pathway in the underlying mechanism.

**Methods:**

DBA/1 mice with collagen II-induced arthritis were treated with GMSCs from the C57BL/6 J mouse, the B6Smn.C3-FasL^gld^/J mouse (FasL^–/–^ GMSCs), and FasL overexpressed FasL^–/–^ GMSCs (FasL TF GMSCs). Inflammation was evaluated by measuring clinical score, tumor necrosis factor (TNF)-α and anti-collagen II antibody levels, and histological analyses. The levels of CD4^+^ Th cell subsets in spleens and draining lymph nodes were assessed by flow cytometric analysis.

**Results:**

Systemic infusion of GMSCs can significantly reduce the severity of experimental arthritis, and resume the balance of Th cell subsets. FasL^–/–^ GMSCs failed to induce apoptosis of activated T cells in vitro and in vivo, and therefore show no therapeutic effects, whereas FasL TF GMSCs can rescue the immunosuppressant effects in the treatment of CIA.

**Conclusions:**

GMSC-based therapy induces T-cell apoptosis via the FasL/Fas pathway and results in immune tolerance and amelioration of the CIA inflammation.

## Background

Rheumatoid arthritis (RA) is a chronic, progressive, and inflammatory autoimmune disease involved in multiple systems, and it primarily affects the small arthrodial joints [1, 2]. Pathologically, the hyperproliferation of the synovial membrane and accumulation of activated T cells and macrophages leads to cartilage degradation and erosion of bone in the joints [1]. The precise etiology of RA is still unknown. Extensive evidence suggests it is the result of interactions between genes and environmental factors [3]. The breakdown of the immunologic self-tolerance results in aberrant immune responses directed at self-antigens in the joints. Antigen-driven T cells and B cells are thought to participate in the rheumatoid process. The therapeutic interventions aim to modulate pathogenic cells, neutralize the effector molecules, and restore tolerance. However, current drug therapy for RA is still not curative [1].

Mesenchymal stem cells (MSCs) have been isolated from almost every tissue. They possess the capacity for self-renewal and multipotent differentiation into multiple mesenchymal lineages, including bone, fat, and cartilage [4, 5]. Moreover, MSCs display anti-inflammatory and immunomodulatory capacities, both in vitro and in vivo [6, 7]. Recent studies have demonstrated that MSCs could also be isolated from gingivia tissue (GMSCs), and that they exhibit many advantages over bone marrow-derived MSCs (BMSCs). GMSCs are easy to access from the dental clinic, and they are more morphologically and functionally stable, and hence less tumorigenic. They proliferate rapidly while remaining homogenous. Transplantation of GMSCs has been proven to have therapeutic effects on experimental colitis [8, 9] and mitigating chemotherapy-induced oral mucositis [10], as well as in autoimmune arthritis in mouse models [11].

Collagen-induced arthritis (CIA) is an animal model frequently used to study the effect of new therapeutics for RA, and it shares several clinical, histological, immunological, and genetic features with human RA [12]. In this study, we attempt to test whether one-time transplantation of GMSCs could ameliorate CIA. Our findings suggest that GMSC-mediated T-cell apoptosis via a FasL/Fas pathway results in immune tolerance and ameliorates the severity of CIA in mice.

## Methods

### Animals

DBA/1 J mice (all male, aged 6–8 weeks), C57BL/6 J, and B6Smn.C3-FasL^gld^/J (BL6 *gld*) mouse lines (male and female, aged 6–8 weeks) were purchased from the Jackson Lab. The *gld* strain has spontaneous mutations in FasL, with no other spontaneous mutations. All experiments using mice were performed in accordance with protocols (University of Southern California #10941) approved by the Institutional Animal Care and Use Committee at the University of Southern California.

### Antibodies

Anti-Sca1-PE, CD44-PE, CD73-PE, CD90-PE, CD34-PE, CD45-PE, CD4-PerCP, CD25-APC, CD3e, and CD28 antibodies were purchased from BD Biosciences (San Jose, CA, USA). Anti-CD105-PE, Foxp3-PE, IL17-PE, and IFNγ-APC antibodies were purchased from eBioscience (San Diego, CA, USA). Anti-FasL antibody was purchased from Santa Cruz Biotechnology (Dallas, TX, USA). Anti-β-Actin antibody was purchased from Sigma-Aldrich (St. Louis, MO, USA).

### Isolation, culture, and differentiation of mouse GMSCs

C57BL/6 J and B6Smn.C3-FasL^gld^/J mice were used at the age of 8 weeks for donation of GMSCs. GMSCs were isolated as described by Xu et al. [9]. GMSCs (passage 3) were cultured at 37 °C in 5 % CO_2_ using α-MEM (Invitrogen, Grand Island, NY, USA) supplemented with 20 % fetal bovine serum (FBS; Invitrogen) and penicillin/streptomycin (Invitrogen).

For GMSC surface molecule analysis, the cells were harvested and stained with PE-conjugated monoclonal antibody against CD44, CD90, CD73, Sca1, CD34, CD45 (BD Biosciences), and CD105 (eBioscience), followed by analyzing with FACS Calibur flow cytometry.

For osteogenic induction, GMSCs were cultured in medium containing 2 mM β-glycerophosphate (Sigma-Aldrich), 100 μM L-ascorbic acid 2-phosphate, and 10 nM dexamethasone (Sigma-Aldrich). After 4 weeks of induction, the cultures were stained with alizarin red for mineralized nodule formation.

For adipogenic induction, 500 nM isobutylmethylxanthine (Sigma Aldrich), 60 μM indomethacin (Sigma-Aldrich), 500 nM hydrocortisone (Sigma Aldrich), 10 μg/ml insulin (Sigma-Aldrich), and 100 nM L-ascorbic acid phosphate were added into the growth medium. After 14 days, the cultured cells were stained with Oil Red-O (Sigma Aldrich), and positive cells were quantified under microscopy.

### Overexpression of FasL

The 293T cells for lentivirus production were seeded in a 10-cm culture dish until 80 % confluence was reached. Plasmids with the proper proportion (fasl gene expression vector: psPAX: pCMV-VSV-G (Addgene) = 5:3:2) were mixed in opti-MEM (Invitrogen) with Lipofectamin LTX (Invitrogen) according to the protocol of the manufacturer. EGFP expression plasmid (Addgene) was used as a control. The supernatant was collected at 48 h after transfection and filtered through a 0.45-μm filter to remove cell debris. For infection, the supernatant containing lentivirus was added into the target cell culture in the presence of 4 μg/ml polybrene (Sigma), and the transgene expression was validated by green fluorescent protein (GFP) under microscopic observation.

### Induction and treatment of CIA

For CIA induction, the DBA/1 mice were immunized intradermally at the base of the tail with 100 μg chicken collagen type II (CII; Chondrex, Redmond, WA, USA) emulsified in complete Freund’s adjuvant (CFA; Chondrex), followed by a booster immunization with 100 μg CII in incomplete adjuvant (Chondrex) (Fig. 2a).

At the moment of the boost (day 21), passage three GMSCs of C57BL/6 J mouse (GMSC-WT), B6Smn.C3-FasL^gld^/J mouse (FasL^–/–^ GMSCs), and FasL overexpressed FasL^–/–^ GMSCs (FasL TF GMSCs) were infused (1 × 10^6^ cells) into CIA mice (*n* = 6) via the lateral tail vein. In the control group, mice received phosphate-buffered saline (PBS) infusion (*n* = 6).

Mice were monitored twice weekly for signs of arthritis based on paw swelling and arthritis scores. Clinical arthritis was evaluated using the following scale: 0 = no damage; 1 = paw with detectable swelling in a single digit; 2 = paw with swelling in more than one digit; 3 = paw with swelling of all digits and instep; and 4 = severe swelling of the paw and ankle.

At the end of the experiments (day 56), we killed the animals and collected peripheral blood, draining lymph nodes (DLNs), spleenocytes, and limbs for further studies.

The animal experiments were performed in three independent experiments.

### Flow cytometric analysis

Spleen and DLN cells were collected from CIA mice; 1 × 10^6^ spleen/DLN cells were incubated with 1 μg anti-CD4 antibody for 30 min on ice under dark conditions. For regulatory T cell (Treg) analysis, 1 μg anti-CD25 was added during the incubation. After cell fixation and permeabilization using a Foxp3 staining buffer kit (eBioscience), cells were stained with 1 μg anti-Foxp3 for Tregs and anti-IFN-γ/anti-IL17 for Th1 and Th17. After washing with FACS buffer (PBS plus 0.4 % bovine serum albumin (BSA)), cells were analyzed using a FACS Calibur flow cytometer with FlowJo software.

### T-lymphocyte apoptosis assay

WT GMSCs, FasL^-/-^ GMSCs, or FasL overexpressed FasL TF GMSCs (2 × 10^5^) were seeded on a 24-well culture plate (Corning) containing Dulbecco’s modified Eagle’s medium (DMEM; Lonza, Basel, Switzerland) with 10 % heat-inactivated FBS, 50 μM 2-mercaptoethanol, 10 mM HEPES, 1 mM sodium pyruvate (Sigma-Aldrich), 1 % non-essential amino acid (Cambrex, East Rutherford, NY, USA), 2 mM L-glutamine, 100 U/ml penicillin, and 100 mg/ml streptomycin. After incubation for 24 h, T lymphocytes (1 × 10^6^) from the spleen, pre-stimulated with plate-bound anti-CD3e (3 μg/ml) and soluble anti-CD28 (2 μg/ml) antibodies, were directly loaded onto GMSCs and co-cultured for 2 days. Apoptotic T cells were detected by staining with CD3 antibody, followed by the AnnexinV Apoptosis Detection Kit I (BD Bioscience), and then analyzed by FACS Calibur flow cytometer.

### Enzyme-linked immunosorbent assay

The sera of the CIA mice in GMSC-treated and untreated groups were isolated and frozen at −80 °C. The levels of cytokines (measured as pg/100 μl in six independent experiments) and anti-CII antibody (measured as KU/100 μl in six independent experiments) were determined by enzyme-linked immunosorbent assay (ELISA) using commercially available kits. Mouse tumor necrosis factor (TNF)-α (Bioscience) and mouse anti-collagen II antibody Kits (Cayman, USA) were used according to the manufacturer’s instructions.

### Western blot analysis

Total protein was extracted using M-PER mammalian protein extraction reagent (Thermo, Rockford, IL). Samples (20 μg) were applied and separated on 10 % NuPAGE gel (Invitrogen), followed by transferring to nitrocellulose membranes (Millipore Inc.). Membranes were blocked in 5 % non-fat dry milk and 0.1 % Tween-20 for 1 h, followed by incubation overnight with primary antibody against mouse FasL (Santa Cruz Biotechnology) diluted at 1:1000 in blocking solution. HRP-conjugated secondary antibody (Santa Cruz Biotechnology) at a dilution of 1:10,000 was used to treat the membranes for 1 h. Immunoreactive proteins were detected using SuperSignal West Pico Chemiluminescent Substrate (Thermo) and BioMax film (Kodak, Rochester, NY, USA).

### Histologic analyses

Four percent paraformaldehyde-fixed limbs were decalcified and paraffin-embedded using standard histologic techniques. Serial 6-μm sections were cut and stained with hematoxylin and eosin to examine morphologic features and assess the histologic arthritis score. Sections were analyzed microscopically for the degree of inflammation and for cartilage and bone destruction according to the method reported previously [11], using the following scale: 0 = normal synovium; 1 = synovial membrane hypertrophy and cell infiltrates; 2 = pannus and cartilage erosion; 3 = major erosion of cartilage and subchondral bone; and 4 = loss of joint integrity and ankylosis.

### Statistics

SPSS 13.0 was used to perform statistical analysis. Significance was assessed by one-way analysis of variance (ANOVA) followed by a Newman–Keuls post hoc test. *P* values less than 0.05 were considered as significant.

## Results

### Isolation and characteristics of mouse GMSCs

**Fig. 1:**
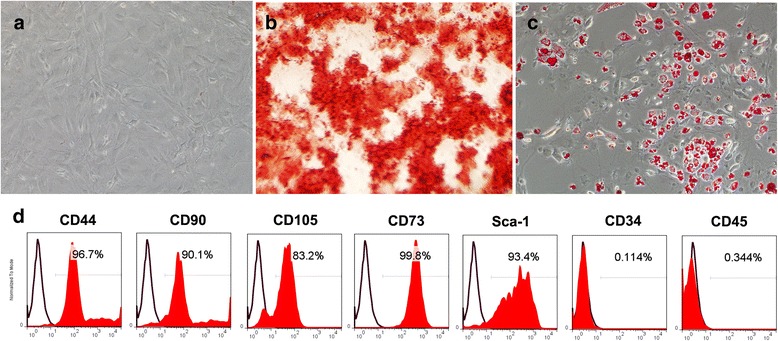
GMSC characterization. **a** Cell culture of passage 3. Original magnification × 40. **b** Alizarin red staining showed the mineralization potential of GMSCs after osteogenic differentiation. Original magnification × 40. **c** Oil red staining showed the adipogenic potential of GMSCs after adipogenic differentiation. Original magnification × 40. **d** Flow cytometric analysis showed that GMSCs were positive for the surface molecules CD44, CD90, CD105, CD73, and Sca1, while negative for CD34 and CD45

GMSCs were successfully isolated from C57BL/6 J mice. They exhibited a characteristic fibroblastic morphology. Under appropriate conditions they could be differentiating into adipocytes and osteocytes. They were negative for CD34, and CD45, but positive for CD44, CD90, CD105, CD73, and Sca1 (Fig. 1).

### A single intravenous injection of GMSCs is very effective in reversing inflammation on CIA

**Fig. 2:**
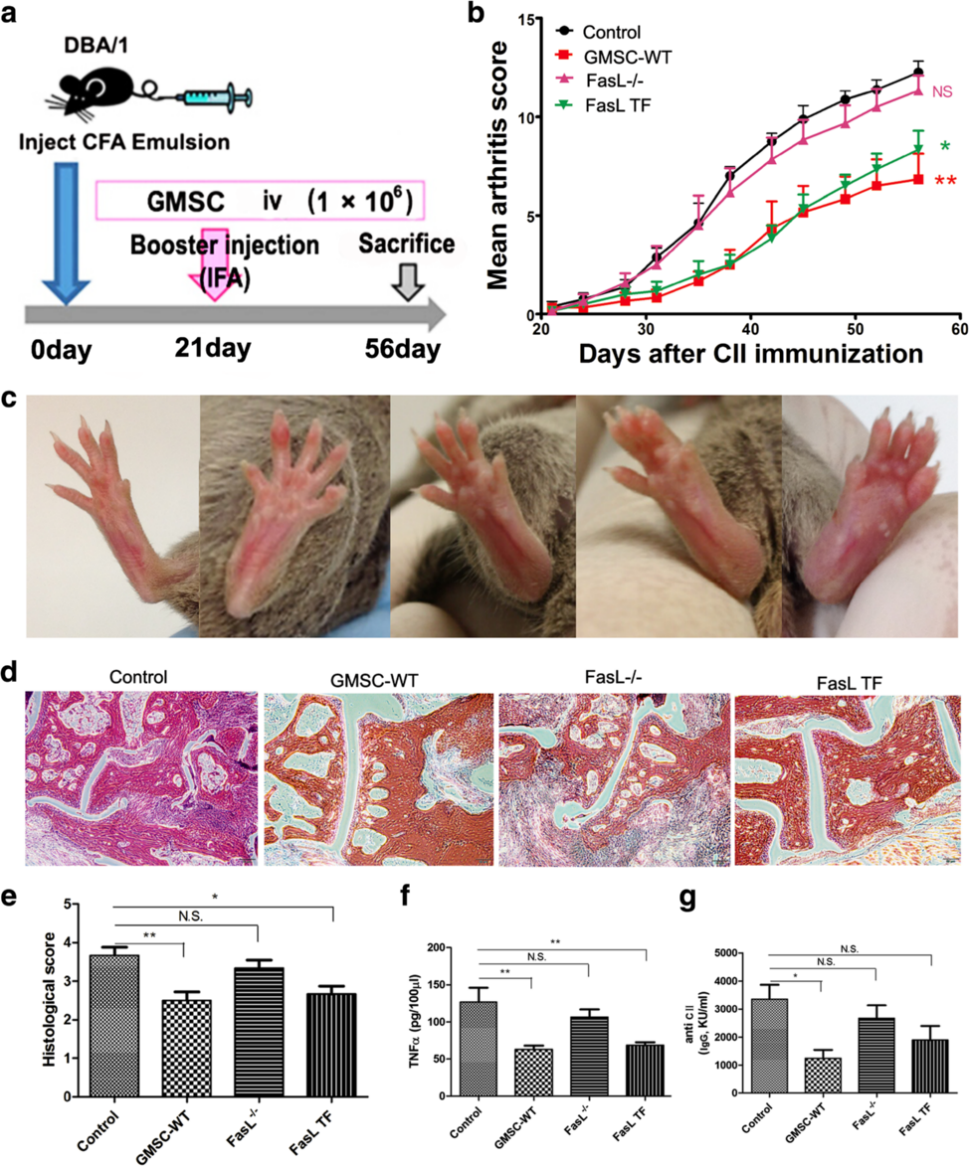
Gingival-derived mesenchymal stem cells (*GMSCs*) attenuate the inflammatory responses in collagen-induced arthritis (CIA) mice via a Fas/FasL pathway. **a** Schema showing GMSC infusion in CIA mice. **b** Arthritis severity scores were determined at various time points after immunization. **c** Arthritis severity score system using a 5-point scale: representative images of swollen hind paws ranging from normal (*left*) to grossly swollen (*right*). **d**, **e** Histological analysis of paw joints of PBS (*Control*), GMSC derived from WT mice (*GMSC-WT*), FasL^–/–^ GMSC- (*FasL* ^*–/–*^), and FasL TF GMSC (*FasL TF*)-treated mice using hematoxylin and eosin staining (original magnification × 40) and score. Serum concentrations of **f** tumor necrosis factor α (*TNFα*) and **g** anti-CII IgG, analyzed by ELISA; *n* = 6 in each group. The animal experiments were performed in three independent experiments. *P < 0.05, **P < 0.01. Values are given as mean ± SEM. *CFA* complete Freund’s adjuvant, *CII* collagen type II, *N.S.* not significant

In this study, all four groups of immunized DBA/1 mice showed signs of CIA (*n* = 6 for each group). Previous scholars proved that MSCs were able to suppress immune reactions in a non-MHC-restricted manner, and allogeneic MSCs might be more effective in clinical application [13]. Hence, we used allogeneic GMSCs of C57Bl/6 mice as immunosuppressant agents for CIA (DBA/1 mice). The present study demonstrated that GMSCs derived from wide-type mice (GMSC-WT) had immunotherapeutic effects on the CIA mice. The arthritis score was significantly reduced compared with that of the PBS control group after GMSC infusion (Fig. 2). The histopathologic analysis indicated that the GMSC-treated mice, as determined on day 35 post-GMSC infusion, exhibited a lower degree of inflammation (synovitis), pannus formation, bone, and cartilage destruction than PBS-treated mice, as assessed by the historical activity index (Fig. 2d). Treatment with GMSCs also reduced the serum levels of CII-specific IgG and the proinflammatory cytokine TNF-α (Fig. 2f and g).

### The FasL/Fas pathway played an important role in GMSC-mediated T-cell responses in CIA mice

A previous study has demonstrated that FasL plays an important role in GMSC-mediated therapy in mice with dextran sulfate sodium (DSS)-induced colitis by inducing immune tolerance [9]. Therefore, we hypothesized that the FasL/Fas signaling pathway has a similar contribution to the immunoregulatory function of GMSCs in the treatment of CIA mice.

Figure 2 shows that the FasL^–/–^ GMSCs exhibited no protective effect on the CIA mouse model, whereas FasL TF GMSCs can recover the therapeutic function and downregulate the inflammatory response. The difference between the PBS-treated (control) and the FasL^–/–^ GMSC-treated mice did not achieve statistical significance, as assessed by arthritis score, historical score, and serum TNF-α concentration, whereas FasL TF GMSC transplantation significantly decreased the values of the above parameters (apart from serum anti-CII tilts), which are important indexes for the severity of the CIA.

**Fig. 3:**
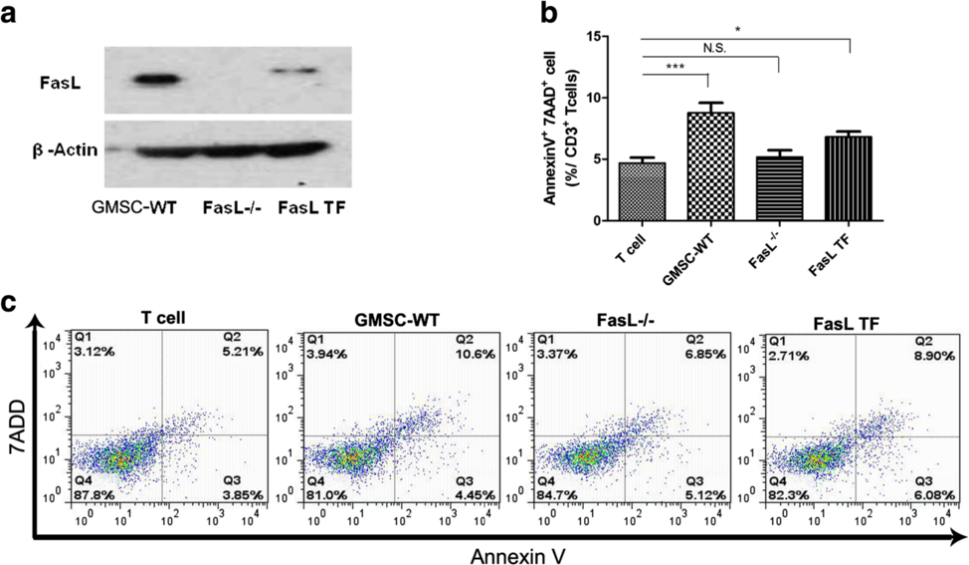
In vitro co-culture system confirmed that FasL expression affects the immunomodulatory properties of GMSC. **a** Comparison of FasL expression in WT GMSCs (*GMSC-WT*), gldGMSCs (*FasL* ^*–/–*^), and FasL TF GMSCs (*FasL TF*). **b**, **c** When co-cultured with the T cells, apoptosis assay showed a decreased capacity of FasL^–/–^ GMSCs to induce AnnexinV^+^7AAD^+^ double-positive apoptotic T cells compared with the WT GMSCs, whereas FasL TF GMSCs can regain the immunomodulatory properties. The results were representative of three independent experiments. *P < 0.05, ***P < 0.001. Values are given as mean ± SEM. *N.S.* not significant

Next, we used a GMSC/T-cell co-culture system to show that FasL^–/–^ GMSCs had a decreased capacity to induce AnnexinV^+^7AAD^+^ double-positive apoptotic T cells when compared to WT GMSCs, whereas FasL TF GMSCs can regain the immunomodulatory properties (Fig. 3). These findings confirmed that FasL expression affects the immunosuppressive properties of GMSCs.

### GMSC transfer therapy influences the polarization of the Th cells

**Fig. 4:**
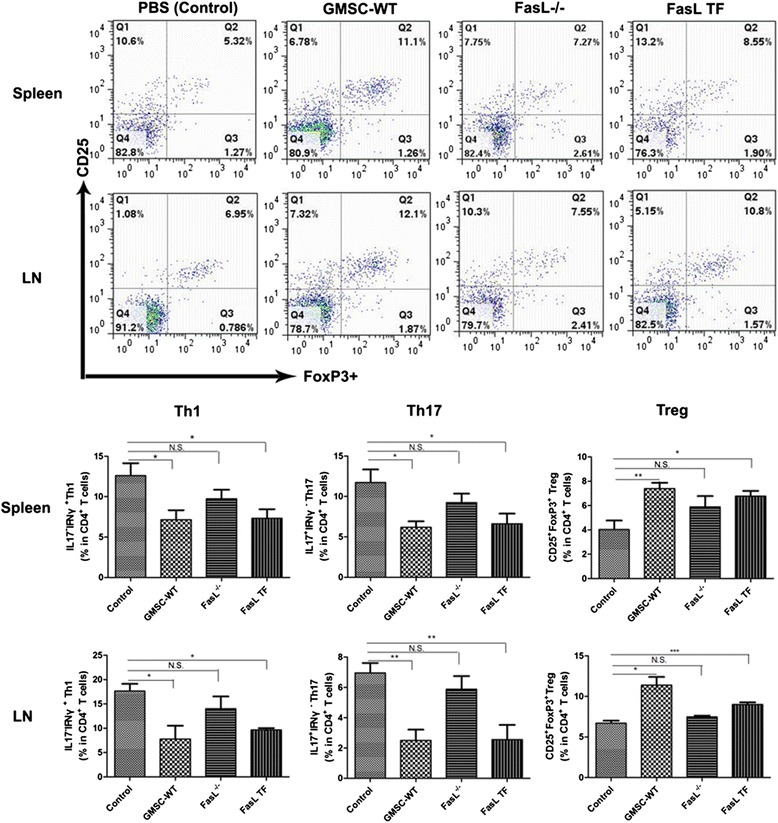
GMSC transfer therapy influences the polarization of the Th cells. **a** Representative flow cytometry data show the expression of CD25 and FoxP3 gated on CD4 cells (regulatory T cells; *Tregs*) in the spleens and DLNs of four groups of CIA mice. **b** WT GMSCs (*GMSC-WT*) and FasL TF GMSCs (*FasL TF*) significantly decreased the percentage of CD4^+^INF-γ^+^ Th1 and CD4^+^IL-17^+^ Th17 cells in the mouse DLN and spleen, and increased the percentage of CD4^+^CD25^+^FoxP3^+^ Treg, whereas FasL^–/–^ GMSCs (*FasL* ^*–/–*^) did not significantly alter the frequencies of the subset Th cells in the CIA mice. *n* = 6 in each group.*P < 0.05, **P < 0.01. Values are given as mean ± SEM. *IL* interleukin, *LN* lymph node, *N.S.* not significant

Flow cytometric analysis of CD4^+^ Th cells shows that WT GMSCs and FasL TF GMSCs can significantly decrease the percentage of CD4^+^INF-γ^+^ Th1 and CD4^+^IL-17^+^ Th17 cells, and increase the percentage of CD4^+^CD25^+^FoxP3^+^ regulatory T cells (Tregs) in the DLN and spleen of the CIA mice, whereas FasL^–/–^ GMSCs did not significantly alter the frequencies of the subset Th cells in the CIA mice (Fig. 4).

## Discussion

In recent years, more and more interest has been taken on the immunomodulating property of MSCs. The immunosuppressive capacities of MSCs were evaluated in experimental animal models, as well as in humans, to treat autoimmune diseases [13–16]. In this study, we isolated and characterized MSCs from the gingival tissues of the mouse, and tested their therapeutic effects using CIA mouse models. We confirmed that GMSCs exhibited similar stem cell properties and immunomodulatory properties as BMSCs. They express a similar profile of surface markers, and exhibit multipotent differentiation into different cell lineages, including mesodermal (adipocytes and osteocytes).

CIA is a reliable model of RA which is widely used in the development of therapeutics. Augello et al. [17] first reported that a single injection of mouse BMSCs prevented the occurrence of severe, irreversible damage to bone and cartilage in CIA mice. We demonstrated that systemic infusion of allogeneic mouse GMSCs also can efficiently ameliorate both clinical and histopathological severity of the CIA inflammation. The clinical score decreased by 44 % on average, and the histological score by 32 %. The serum levels of TNF-α and CII-specific IgG decreased remarkably by 50 % and 63 %, respectively, which further confirmed that GMSC therapy could downregulate the immune response and reduce tissue damage. More recently, several scholars reported that rat BMSCs [18], human AD-MSCs [19], and human GMSCs [11] were also beneficial for CIA. Previous studies demonstrated that MSCs could overcome MHC mismatch; when xenogeneic, allogeneic, or syngeneic MSCs were utilized, each of them may exhibit a therapeutic effect [20]. Although the precise underling mechanism remains to be elucidated, a variety of factors, including transforming growth factor (TGF)-β, interleukin (IL)-10, prostaglandin E2 (PGE2), nitric oxide (NO), indoleamine 2,3-dioxygenase (IDO), and FasL have been identified as potential regulators of MSC-based immunomodulation [21, 22].

It is well known that T cells play a key role in induction, maintenance, and relapse of RA, and they are an important target for the development of new anti-arthritic therapies [23]. Previous scholars have regarded RA as a Th1-driven disease, with a predominance of Th1 cytokines and a lack of Th2 cytokines, whereas in recent years the role of Th17 has been emphasized in joint inflammation and destruction [11]. Tregs are involved at the center of immunosuppressive reactions, and suppress several autoreactive responses and maintain self-tolerance in the immune system. Augello et al. [17] found that MSC viability was not required for their long-term immunosuppressant action, and that the prolonged immunosuppressive activity of MSCs could be attributed to the action of Treg clones that can be activated by an antigen-specific stimulus. We found the proportion of Th1 and Th17 to be elevated in CIA mice, along with a reduced level of Treg in CD4^+^. GMSC transplantation can upregulate the Treg level (by ≈ 84 % in spleen, and ≈ 70 % in DLN) and reduce the Th1 and Th17 level, leading to immune tolerance and thereby ameliorating severity of the inflammation. The GMSC/T cell co-culture experiments further reveal that GMSCs could induce apoptosis of activated T cells (Fig. 3). Therefore, GMSCs are a plausible cell source for MSC-based therapy in RA by rescuing the T-cell homeostasis of the recipients.

FasL is a type II transmembrane protein that belongs to the tumor necrosis factor (TNF) family. Its binding with its receptor induces apoptosis. FasL/Fas interactions play an important role in the regulation of the immune system [22, 24]. Previous scholars demonstrated that MSCs (BMSCs or SHED) may induce T-cell apoptosis, which further upregulates the Tregs via a high level of macrophage-released TGF-β, and results in immune tolerance [22, 25]. In this study, we isolated FasL mutant GMSCs from B6Smn.C3-FasL^gld^/J mice (FasL^-/-^ GMSC). We found that WT GMSCs, but not FasL^-/-^ GMSCs, elevate the Treg levels and reduce the proportion of the Th1 and Th17 subsets, whereas FasL TF GMSCs can rescue the immunosuppressant effects in the treatment of CIA, as well as in vitro co-culture system. Therefore, FasL-induced T-cell apoptosis is required for GMSC-based cell therapy for CIA. Figure 2g shows that the decrease in serum anti-CII tilts in the FasL TF GMSC-treated group has not reach statistical significance (*P* > 0.05), which may be due to the small sample size. Moreover, the direct/indirect effect of FasL on the humeral immunity may be different from that on the cell-mediated immunity, and the changes in the corresponding indexes are not always synchronized. Figure 3a suggests that, although the FasL level of the FasL TF GMSCs is eminently lower than that of the WT GMSCs, they exhibit similar therapeutic potential. Previous scholars have shown that MSCs can inhibit, in a dose-dependent manner, the proliferation of, and cytokine production by, T cells, B cells, natural killer cells, and dendritic cells via multiple mechanisms; there were also a few studies which failed to demonstrate any improvement in experimental CIA with MSC treatment [26]. The conflicting results emphasize the need for standardizing each step of the MSC therapy. Further studies are required to find the best GMSC dose, and the dose-related effects of GMSCs, as well as the FasL level, should be reassessed in a more standardized manner to achieve the best therapeutic outcomes.

## Conclusions

Taken together, the present study demonstrates that systemic infusion of allogeneic mouse GMSCs can efficiently ameliorate severity of the CIA inflammation by inducing T-cell homeostasis and immune tolerance. FasL plays an important role in the mechanism underlying the beneficial effect of the GMSC-based cell therapy.

## Abbreviations

BMSC: Mesenchymal stem cell from bone marrow
CFA: Complete Freund’s adjuvant
CIA: Collagen-induced arthritis
CII: Collagen type II
DLN: Draining lymph node
DSS: Dextran sulfate sodium
ELISA: Enzyme-linked immunosorbent assay
FBS: Fetal bovine serum
GFP: Green fluorescent protein
GMSC: Mesenchymal stem cell derived from gingival tissue
IDO: Indoleamine 2,3-dioxygenase
IFN: Interferon
IL: Interleukin
MSC: Mesenchymal stem cell
NO: Nitric oxide
PBS: Phosphate-buffered saline
PGE2: Prostaglandin E2
RA: Rheumatoid arthritis
SHED: Stem cells from human exfoliated deciduous teeth
TGF: Transforming growth factor
TNF: Tumor necrosis factor
Treg: Regulatory T cell
WT: Wild-type
